# Synergistic Effect of History of Cardiovascular Disease and Mental Distress on Post-Traumatic Stress Disorder after the Great East Japan Earthquake: The Fukushima Health Management Survey

**DOI:** 10.3390/ijerph181910283

**Published:** 2021-09-29

**Authors:** Kazuhide Tezuka, Yasuhiko Kubota, Tetsuya Ohira, Hironori Nakano, Masaharu Maeda, Hirooki Yabe, Seiji Yasumura, Mayumi Harigane, Yuji Shimizu, Takeo Okada, Masahiko Kiyama, Kenji Kamiya, on behalf of the Fukushima Health Management Survey Group

**Affiliations:** 1 Department of Cardiovascular Disease Prevention, Osaka Center for Cancer and Cardiovascular Disease Prevention, Osaka 536-0025, Japan; kubota@osaka-ganjun.jp (Y.K.); shimizu@osaka-ganjun.jp (Y.S.); tokada@osaka-ganjun.jp (T.O.); kiyama@osaka-ganjun.jp (M.K.); 2 Department of Epidemiology, School of Medicine, Fukushima Medical University, Fukushima 960-1295, Japan; teoohira@fmu.ac.jp (T.O.); h-nakano@fmu.ac.jp (H.N.); 3 Radiation Medical Science Center for the Fukushima Health Management Survey, Fukushima Medical University, Fukushima 960-1295, Japan; masagen@fmu.ac.jp (M.M.); harigane@fmu.ac.jp (M.H.); kkamiya@hiroshima-u.ac.jp (K.K.); 4 Department of Disaster Psychiatry, School of Medicine, Fukushima Medical University, Fukushima 960-1295, Japan; 5 Department of Neuropsychiatry, School of Medicine, Fukushima Medical University, Fukushima 960-1295, Japan; hyabe@fmu.ac.jp; 6 Department of Public Health, School of Medicine, Fukushima Medical University, Fukushima 960-1295, Japan; yasumura@fmu.ac.jp; 7 Research Institute for Radiation Biology and Medicine, Hiroshima University, Hiroshima 734-8553, Japan

**Keywords:** the Great East Japan Earthquake, cardiovascular disease, mental distress, post-traumatic stress disorder, the relative excess risk due to interaction, attributable proportion

## Abstract

Cardiovascular disease (CVD) and mental distress have been suggested to be associated with post-traumatic stress disorder (PTSD), but the effect of their combination on PTSD is unknown. We reviewed the synergistic effects of the history of CVD and mental distress on the possibility of PTSD among residents in Fukushima after the Great East Japan Earthquake. This cross-sectional study was conducted among 38,392 participants aged 40–74 years in the evacuation area who applied for the Fukushima Health Management Study in Fiscal Year 2011. Relative excess risk due to interaction (RERI), attributable proportion (AP), odds ratio (OR), and 95% confidence interval (CI) were calculated to investigate the combined effect of history of CVD and mental distress on PTSD. We identified 8104 probable cases of PTSD (21.1%). History of CVD, mental distress, and their combination were positively associated with probable PTSD: the multivariable ORs (95% CIs) were 1.44 (1.04, 2.01), 20.08 (18.14, 22.22), and 26.60 (23.07, 30.67), respectively. There was a significant increase in RERI: the corresponding RERI (95% CI) and AP were 6.08 (3.16, 9.00) and 22.9%. Gender-specific analyses showed similar associations. Thus, we found a supra-additive association of history of CVD and mental distress with probable PTSD after the disaster.

## 1. Introduction

Post-traumatic stress disorder (PTSD) is one of the leading mental illnesses after extremely traumatic events. According to a review article, the prevalence of PTSD among direct victims of man-made disasters was around 25% to 75%, and the prevalence after natural disasters was 5% to 60% [1]. Earthquake, a large-scale traumatic event over communities, is a natural trigger for the higher burden of PTSD. The prevalence of PTSD following earthquake was estimated to be 28.8% [2]. It is often impossible to provide cognitive intervention for all residents after an earthquake, which has been promising to prevent individuals from chronic PTSD after a disaster [3]. Therefore, it is necessary to identify the groups associated with high PTSD, who are particularly in need of therapeutic resources.

Cardiovascular disease (CVD) and mental distress are considered risk factors for PTSD. In a longitudinal study in an Asian community, the history of CVD and major depressive disorder was associated with future PTSD [4]. Both CVD and depression are related to autonomic dysregulation, which seems to induce vulnerability to traumatic stress [5,6]. Several psychological related factors have also been suggested to interact with each other concerning PTSD. Negative affect and peritraumatic dissociation had a synergistic effect in predicting the severity of PTSD symptoms in a sample of Brazilian police officers [7]. However, only a few studies have shown an interaction between the physical and psychological factors (such as CVD history and mental distress) related to PTSD after an earthquake. Thus, we investigated the synergistic effect of the history of CVD and mental distress on the possibility of PTSD among residents in the evacuation area of Fukushima after the Great East Japan Earthquake. We used the PTSD symptoms questionnaire to assess PTSD instead of diagnosis by a physician and adopted the phrase “probable PTSD”.

## 2. Materials and Methods

### 2.1. Study Population

A cross-sectional study was conducted on the survey population. The study population included 42,823 residents (19,591 men and 23,232 women) from the evacuation area who were 40–74 years old and applied for the Mental Health and Lifestyle Survey of the Fukushima Health Management Study in the fiscal year 2011. Details of the Fukushima Health Management Study protocol have been described elsewhere [8,9]. The zone consisted of Hirono Town, Naraha Town, Tomioka Town, Kawauchi Village, Okuma Town, Futaba Town, Namie Town, Katsurao Village, Minamisoma City, Tamura City, Yamakiya district of Kawamata Town, and Iitate Village. Four thousand, four hundred, and thirty-one (4431) participants (1658 men and 2773 women) with missing data on the history of CVD, mental distress, or traumatic symptoms were excluded from the study, leaving 38,392 participants (17,933 men and 20,459 women) for analysis.

All participants received an invitation letter explaining the purpose of the survey. It clearly stated that the response to the survey questionnaires would be regarded as providing their consent for participation. The study protocol was approved by the Ethics Review Committees at Fukushima Medical University (Number 1316) and Osaka Center for Cancer and Cardiovascular Disease Prevention (Number 30-Rinri-16). The study was conducted in accordance with the World Medical Association Declaration of Helsinki.

### 2.2. Measurement for Physical and Mental Health Status

Probable PTSD was measured using the PTSD Checklist—Stressor-Specific Version (PCL-S) [10]. It consisted of 17 items for detecting PTSD, and each item was scored with a 5-point Likert scale. PCL-S score was calculated by summing the scores for each item ranging from 17 to 85. Previously, we reported the scale’s reliability and validity in the Fukushima Health Management Survey [11,12]. Probable PTSD was classified as PCL-S score ≥44 [10].

Mental distress was assessed using a Kessler 6 (K6) scale [13]. It contained six items on common psychological distress, rated on five levels. The K6 score is the sum of scores for item scores ranging from 0 to 24. The Japanese version of K6 demonstrated screening performance substantially equivalent to those reported with the original English versions [14]. Mental distress was classified by the K6 score of ≥5 and included moderate to severe mental distress necessitating mental health treatment [15].

The self-rating questionnaire was used to assess the participants’ history of CVD including ischemic heart disease (myocardial infarction and angina pectoris). It also reported on ischemic stroke and hemorrhagic stroke (intracerebral and subarachnoid hemorrhage). Smoking status (never, former, current, and missing), alcohol intake status (never, former, current, and missing), physical activity (almost never, once/week, 2–4 times/week, almost every day, and missing) and history of hypertension, diabetes mellitus, and dyslipidemia (no, yes and missing) were evaluated in the same way.

### 2.3. Statistical Analyses

The characteristics of the participants were assessed using a combination of CVD history and mental distress. A multivariate logistic regression model was used to examine the odds ratio (OR) and 95% confidence interval (CI) of likely combinations of PTSD. The model included probable PTSD as dependent variable; combination of CVD history and mental distress (without CVD history/without mental distress, with CVD history/without mental distress, without CVD history/with mental distress and with CVD history/with mental distress) as independent variable; and age, gender, smoking status, alcohol intake status, physical activity and history of hypertension, diabetes mellitus, and dyslipidemia as confounding variables.

To test for additive interactions (supra-additive associations) among the history of CVD, mental distress, and probable PTSD, the relative excess risk due to interactions (RERI) was calculated with ORs for (1) with CVD history/with mental distress; (2) with CVD history/without mental distress; and (3) without CVD history/with mental distress [16] and the 95% CIs were computed using the delta method [17]. Calculation of the attributable proportions (APs) was conducted RERI/OR (with CVD history/with mental distress) [18]. In the same way, we examined the associations in male and female participants. We also performed the procedure again after confirming the history of CVD subtypes, namely, ischemic heart disease, ischemic stroke, and hemorrhagic stroke.

All statistical tests were two-sided, with *p* < 0.05 regarded as statistically significant. All statistical analyses were performed using SAS version 9.3 (SAS Institute, Inc., Cary, NC, USA).

## 3. Results

### 3.1. Participants’ Characteristics

The participants’ characteristics according to the combination of the history of CVD and mental distress are shown in Table 1. Overall, 2479 (6.5%) participants reported history of CVD and 21,220 (55.3%) had mental distress. Individuals with a history of CVD and mental distress had the highest proportions of history of hypertension, diabetes mellitus, and dyslipidemia, and the lowest proportion for current drinking among the four groups.

### 3.2. Probable PTSD Rate and Its Association

We identified 8104 probable PTSD cases (21.1%). The associations of history of CVD and mental distress with probable PTSD are shown in Table 2. Among all participants, history of CVD, mental distress, and their combination were positively associated with probable PTSD: the multivariable ORs (95% CIs) were 1.44 (1.04, 2.01), 20.08 (18.14, 22.22), and 26.60 (23.07, 30.67), respectively. There was a significant increase in RERI between the history of CVD and mental distress to probable PTSD: the corresponding RERI (95% CI) and AP were 6.08 (3.16, 9.00) and 22.9%. Similar associations were observed among the male and female participants. We performed the same procedures again after specifying the clinical history of CVD subtypes, namely, ischemic heart disease, ischemic stroke, and hemorrhagic stroke (Supplementary Materials Tables S1–S3). A similar association was observed, as shown in Table 2.

## 4. Discussion

We examined how the combination of a history of cardiovascular disease and mental distress in Fukushima residents after the Great East Japan Earthquake was associated with PTSD. We found that this combination was supra-additively associated with possible PTSD. To date, this is the first study to find an additive interaction (i.e., a supra-additive association) between the history of CVD and mental distress to probable PTSD.

Although we cannot currently determine which factors are involved in the interaction, autonomic dysregulation among the participants may be one of the factors. Life-threatening events such as natural disasters could alter the autonomic nervous system (ANS) and activate the sympathetic nervous system (SNS), releasing epinephrine and norepinephrine from the adrenal medulla. This peripheral endocrine system is essential to change the blood flow for fight-or-flight behavior [6]. The ANS dysregulation and SNS hyperactivity after the traumatic events seems to account for some of the PTSD symptomatology. In previous studies, patients with PTSD had sustained urinary norepinephrine and epinephrine elevation [19], increased heart rate and blood pressure [20] and lowered heart rate variability (HRV) [21]. Administration of Yohimbine, leading to norepinephrine release by blocking the α2 adrenergic receptor, induced panic attacks, flashbacks, and higher heart rate and blood pressure in male patients with PTSD [22]. The HRV biofeedback training, in which one tried to maximize HRV, reduced symptoms of PTSD measured by the clinician-administered PTSD Scale and PCL-S in veterans with PTSD [21].

The SNS reactivity to an emotional stressor was suggested to be exacerbated in mentally distressed people. An experimental study on stress indicated that participants with depression and/or anxiety disorder showed significant hyperreactivity of the heart rate and respiratory sinus arrhythmia compared with healthy controls [23]. Prolonged SNS upregulation was presumed to increase cardiovascular events. Furthermore, elevated resting heart rate was linked to sudden cardiac death in the general population; progression of atherosclerosis, ventricular arrhythmias, myocardial ischemia; and plaque disruption in acute coronary syndrome in individuals with coronary heart disease [5]. Psychological distress as well as depression and anger were associated with the increased risk of CVD [5,24,25]. Taken together, both CVD and mental distress may be related to autonomic dysregulation, which induced vulnerability to the traumatic stress.

The study’s strength is the use of many of the residents in the evacuation area after the disaster and participation within approximately one year after the disaster. However, the study also has several limitations. First, because the participation in the research was voluntary, our study did not include people with severe mental disorders of PTSD. Our study also excluded participants with missing data. Therefore, the external validity of this study might decrease. Second, due to being a cross-sectional study, we could not discuss the temporal aspects. Third, the number of participants who had history of hemorrhagic stroke was relatively small. We could not discuss at length the interactions between history of CVD subtypes and mental distress. Fourth, we could not adjust for physical measurements such as body mass index, systolic and diastolic blood pressure, and values from serum examinations due to a lack of data on them. Fifth, this study did not demonstrate the best indicator for selecting therapeutic target group after the disaster.

## 5. Conclusions

We found a supra-additive association of history of CVD and mental distress with probable PTSD among residents in Fukushima after the Great East Japan Earthquake. Individuals with psychological problems complicated by physiological problems such as CVD might be at an increased risk for PTSD after a large-scale disaster.

## Figures and Tables

**Table 1:** Participants’ characteristics according to history of CVD and mental distress.

Characteristics	Without CVDH/Without MD	With CVDH/Without MD	Without CVDH/With MD	With CVDH/With MD	*p*
No. of participants	16242	930	19671	1549	
Age (year), mean (SD)	58.4 (9.4)	64.9 (7.1)	57.6 (9.3)	64.3 (7.2)	<0.001
Men, *n* (%)	8441 (52.0)	679 (73.0)	7898 (40.2)	915 (59.1)	<0.001
Current smoking, *n* (%)	3523 (21.7)	132 (14.2)	4240 (21.6)	236 (15.2)	<0.001
Current drinking, *n* (%)	8534 (52.5)	464 (49.9)	9433 (48.0)	704 (45.5)	<0.001
Daily physical activity, *n* (%)	7840 (48.3)	343 (36.9)	10358 (52.7)	677 (43.7)	<0.001
History of hypertension, *n* (%)	6551 (40.3)	692 (74.4)	8514 (43.3)	1185 (76.5)	<0.001
History of diabetes mellitus, *n* (%)	2844 (17.5)	347 (37.3)	3909 (19.9)	608 (39.3)	<0.001
History of dyslipidemia, *n* (%)	5879 (36.2)	472 (50.8)	8288 (42.1)	909 (58.7)	<0.001

CVD: Cardiovascular disease; CVDH: History of cardiovascular disease; MD: Mental distress; SD: Standard deviation.

**Table 2:** Associations of history of CVD and mental distress with probable PTSD among residents after the earthquake.

Variables	Without CVDH/Without MD	With CVDH/Without MD	Without CVDH/With MD	With CVDH/With MD	RERI (95% CI)	AP (%)
All participants						
No. of participants	16242	930	19671	1549		
Probable PTSD, *n* (%)	423 (2.6)	41 (4.4)	6924 (35.2)	716 (46.2)		
Gender and age adjusted OR (95% CI)	1 (reference)	1.58 (1.14, 2.19)	20.46 (18.49, 22.64)	29.32 (25.49, 33.72)	8.28 (5.11, 11.45)	28.24
Multivariable adjusted OR (95% CI) ^1^	1 (reference)	1.44 (1.04, 2.01)	20.08 (18.14, 22.22)	26.60 (23.07, 30.67)	6.08 (3.16, 9.00)	22.85
Men						
No. of participants	8441	679	7898	915		
Probable PTSD, *n* (%)	210 (2.5)	28 (4.1)	2550 (32.3)	399 (43.6)		
Age adjusted OR (95% CI)	1 (reference)	1.49 (0.99, 2.22)	19.26 (16.66, 22.27)	27.45 (22.69, 33.21)	7.70 (3.78, 11.62)	28.06
Multivariable adjusted OR (95% CI) ^2^	1 (reference)	1.33 (0.89, 2.00)	18.94 (16.37, 21.91)	24.81 (20.42, 30.15)	5.55 (1.92, 9.18)	22.36
Women						
No. of participants	7801	251	11773	634		
Probable PTSD, *n* (%)	213 (2.7)	13 (5.2)	4374 (37.2)	317 (50.0)		
Age adjusted OR (95% CI)	1 (reference)	1.70 (0.96, 3.02)	21.62 (18.76, 24.90)	31.42 (25.52, 38.68)	9.10 (3.88, 14.33)	28.98
Multivariable adjusted OR (95% CI) ^2^	1 (reference)	1.54 (0.87, 2.75)	21.19 (18.39, 24.41)	28.39 (22.99, 35.04)	6.66 (1.89, 11.43)	23.45

AP: Attributable proportion; CI: Confidence interval; CVD: Cardiovascular disease; CVDH: History of cardiovascular disease; MD: Mental distress; OR: Odds ratio; RERI: Relative excess risk due to interaction. ^1^ Adjusted for gender, age, smoking status, alcohol intake status, physical activity status and histories of hypertension, diabetes mellitus, and dyslipidemia. ^2^ Adjusted for age, smoking status, alcohol intake status, physical activity status, and histories of hypertension, diabetes mellitus, and dyslipidemia.

## Data Availability

The datasets analyzed during the present study are not publicly available because the data from the Fukushima Health Management Survey belongs to the government of Fukushima Prefecture and can only be used within the organization.

## Supplementary Materials

The following are available online at https://www.mdpi.com/article/10.3390/ijerph181910283/s1, Table S1: Associations of history of ischemic heart disease and mental distress with probable PTSD among residents after the earthquake, Table S2: Associations of history of ischemic stroke and mental distress with probable PTSD among residents after the earthquake, Table S3: Associations of history of hemorrhagic stroke and mental distress with probable PTSD among residents after the earthquake.
